# hoppet v2 release note

**DOI:** 10.1140/epjc/s10052-025-15200-y

**Published:** 2026-02-16

**Authors:** Alexander Karlberg, Paolo Nason, Gavin Salam, Giulia Zanderighi, Frédéric Dreyer

**Affiliations:** 1 CERN, Theoretical Physics Department, 1211 Geneva 23, Switzerland; 2 INFN, Sezione di Milano-Bicocca, and Universita di Milano-Bicocca, Piazza della Scienza 3, 20126 Milan, Italy; 3 Rudolf Peierls Centre for Theoretical Physics, Clarendon Laboratory, Parks Road, Oxford, OX1 3PU UK; 4 All Souls College, Oxford, OX1 4AL UK; 5 Max-Planck-Institut fur Physik, Boltzmannstr. 8, 85748 Garching, Germany; 6 Physik-Department, Technische Universitat Munchen, James-Franck-Strasse 1, 85748 Garching, Germany; 7 Prescient Design, Genentech, 149 5th Avenue, New York, NY 10010 USA

## Abstract

We document the three main new features in the v2 release series of the hoppet parton distribution function evolution code, specifically support for N$^3$LO QCD evolution in the variable flavour number scheme, for the determination of hadronic structure functions for massless quarks up to N$^3$LO, and for QED evolution to an accuracy phenomenologically equivalent to NNLO QCD. Additionally we describe a new Python interface, CMake build option, functionality to save a hoppet table as an LHAPDF grid and update our performance benchmarks, including optimisations in interpolating PDF tables.

## Introduction

hoppet [1] is a parton distribution function (PDF) evolution code written in modern Fortran, with interfaces also for C/C**++** and earlier dialects of Fortran. It offers both a high-level PDF evolution interface and user-access tof lower-level functionality for operations such as convolutions of coefficient functions and PDFs. It is designed to provide flexible, fast and accurate evolution.

Since the first major release of hoppet, the landscape of PDF evolution codes has evolved substantially, with a range of new open-source codes having been developed, for example the APFEL [2], APFEL**++** [3] and EKO [4] codes supplementing earlier widely-used public codes such as QCDNUM [5] and PEGASUS [6].^1^ Nevertheless, hoppet remains a powerful tool, notably for its ability to reach high and quantifiable accuracies with competitive speed. As a result it often provides a critical reference for benchmark studies, as those presented e.g. in Refs. [8, 9]. It also provides the option of fast (millisecond-scale) evolution with accuracies $\sim 10^{-4}$, which is more than sufficient for phenomenological applications. Furthermore hoppet ’s exposition not just of full PDF evolution (as exploited in Refs. [10–14]), but also of low-level functionality though a stable, public API, has led to its use for a range of fixed-order [15–17], all-order [18–23] and Monte Carlo [24–27] applications. With the advent of ever more precise data from the LHC at CERN, and future very high precision data at the forthcoming Electron-Ion Collider [28] at Brookhaven National Laboratory, the continued development of tools such as hoppet remains important for the field.

This release note documents three major additions to hoppet, made available as part of release 2.0.0: (1) support for QCD evolution at N$^3$LO in the (zero mass) variable flavour number scheme (VFNS) [29] (Sect. 4); (2) support for the determination of massless hadronic structure functions, as initially developed for calculations of vector-boson fusion, and later deep inelastic scattering, cross sections [30–34] (Sect. 5); (3) support for QED evolution (Sect. 6), originally developed as part of the LuxQED project for the evaluation of the photon density inside a proton and its extension to lepton distributions in the proton [35–38].

This release also includes a range of other additions relative to the original 1.1.0 release documented in [1], in particular a Python interface (Sect. 7), a CMake-based build system (Sect. 8), and the ability to write LHAPDF grids (Sect. 9). We have also updated the performance benchmarks and improved the speed for interpolating PDF tables also when loaded from LHAPDF (Sect. 10). hoppet can be obtained by executing 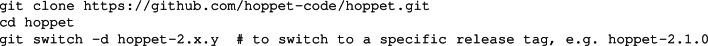 Unified documentation of the whole hoppet package is part of the distribution at https://github.com/hoppet-code/hoppet in the https://github.com/hoppet-code/hoppet/blob/hoppet-2.1.1/docs/manual directory. Details of the other changes since release 1.1.0 can be found in the https://github.com/hoppet-code/hoppet/blob/hoppet-2.1.1/NEWS.md and https://github.com/hoppet-code/hoppet/blob/hoppet-2.1.1/ChangeLog files from the repository.
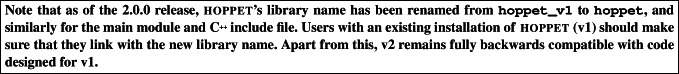


## Perturbative evolution in QCD

**Note:** this section is largely a repetition of Sect. 2 of Ref. [1], and is included again here to help provide context for the discussion that follows in subsequent sections.

First of all we set up the notation and conventions that are used throughout hoppet. The DGLAP equation for a non-singlet parton distribution reads

1 $$\begin{aligned} \frac{\partial q\left( x,Q^2\right) }{\partial \ln Q^2} = \frac{\alpha _s\left( Q^2 \right) }{2\pi }\int _x^1 \frac{dz}{z} P(z,\alpha _s\left( Q^2 \right) ) q\left( \frac{x}{z},Q^2\right) \equiv \frac{\alpha _s\left( Q^2 \right) }{2\pi } P(x,\alpha _s\left( Q^2 \right) ) \otimes q\left( x,Q^2\right) . \end{aligned}$$

The related variable $t\equiv \ln Q^2$ is also used in various places in hoppet. The splitting functions in Eq. (1) are known exactly up to NNLO in the unpolarised case [39–42], and approximately at N$^3$LO [43–56]:

2 $$\begin{aligned} P(z,\alpha _s\left( Q^2 \right) )= &   P^{(0)}(z)+\frac{\alpha _s\left( Q^2 \right) }{2\pi }P^{(1)}(z) + \left( \frac{\alpha _s\left( Q^2 \right) }{2\pi } \right) ^2 P^{(2)}(z) + \left( \frac{\alpha _s\left( Q^2 \right) }{2\pi } \right) ^3 P^{(3)}(z), \, \end{aligned}$$

and up to NNLO [57–62] in the polarised case. The generalisation to the singlet case is straightforward, as it is to the case of time-like evolution,^2^ relevant for example for fragmentation function analysis, where NNLO results are also available [65, 69, 70].

As with the splitting functions, most perturbative quantities in hoppet are defined to be coefficients of powers of $\alpha _s/2\pi$. However there are some places where a different convention is used, either for historical reasons or because external code uses a different convention. In particular the $\beta$-function coefficients of the running coupling equation,

3 $$\begin{aligned} \frac{d\alpha _s}{d\ln Q^2}= &   \beta \left( \alpha _s\left( Q^2 \right) \right) = -\alpha _s(\beta _0\alpha _s+ \beta _1\alpha _s^2 + \beta _2\alpha _s^3 + \beta _3\alpha _s^4) , \end{aligned}$$

are defined internally in hoppet as multiplying powers of $\alpha _s$ directly.

The evolution of the strong coupling and the parton distributions can be performed in both the fixed flavour-number scheme (FFNS) and the variable flavour-number scheme (VFNS). In the VFNS case we need the matching conditions between the effective theories with $n_f$ and $n_{f}+1$ light flavours for both the strong coupling $\alpha _s\left( Q^2 \right)$ and the parton distributions at the heavy quark mass threshold $m_h^2$.

These matching conditions for the parton distributions receive non-trivial contributions at higher orders. In the $\overline{\textrm{MS}}$ (factorisation) scheme, for example, carrying out the matching at a scale equal to the heavy-quark mass, these begin at NNLO^3^: for light quarks $q_{l,i}$ of flavour *i* (quarks that are considered massless below the heavy quark mass threshold $m_h^2$) the matching between their values in the $n_f$ and $n_f+1$ effective theories reads^4^:

4 $$\begin{aligned}&q_{l,i}^{\,\left( n_f+1\right) }\left( x,m_h^2\right) + q_{l,-i}^{\,\left( n_f+1\right) }\left( x,m_h^2\right) = q_{l,i}^{\,(n_f)}\left( x,m_h^2\right) + q_{l,-i}^{\,(n_f)}\left( x,m_h^2\right) \nonumber \\&\qquad + A^{\mathrm{NS,+}}_{qq,h}\left( x\right) \otimes \left( q_{l,i}^{\, (n_f)}\left( x,m_h^2\right) + q_{l,-i}^{\, (n_f)}\left( x,m_h^2\right) \right) + \frac{1}{n_f} \Big \{A^{\textrm{PS}}_{qq,h}\left( x\right) \otimes \Sigma ^{\, (n_f)}\left( x,m_h^2\right) \nonumber \\&\qquad + A^{\textrm{S}}_{qg,h}\left( x\right) \otimes g^{\, (n_f)}\left( x,m_h^2\right) \Big \} \ , q_{l,i}^{\,\left( n_f+1\right) }\left( x,m_h^2\right) - q_{l,-i}^{\,\left( n_f+1\right) }\left( x,m_h^2\right) \nonumber \\&\quad = q_{l,i}^{\,(n_f)}\left( x,m_h^2\right) - q_{l,-i}^{\,(n_f)}\left( x,m_h^2\right) + A^{\mathrm{NS,-}}_{qq,h}\left( x\right) \otimes \left( q_{l,i}^{\, (n_f)}\left( x,m_h^2\right) - q_{l,-i}^{\, (n_f)}\left( x,m_h^2\right) \right) \ , \end{aligned}$$

where $i = 1,\ldots n_f$, while for the gluon distribution, the heavy quark PDF $q_h$, and the singlet PDF $\Sigma \left( x,Q^2\right)$ (defined in Table 1) one has:

5 $$\begin{aligned}&g^{(n_f+1)}\left( x,m_h^2\right) = g^{\, (n_f)}\left( x,m_h^2\right) + A_{\textrm{gq,h}}^{\textrm{S}}(x) \otimes \Sigma ^{(n_f)}\left( x,m_h^2\right) + A_{\textrm{gg,h}}^{\textrm{S}}(x) \otimes g^{(n_f)}\left( x,m_h^2\right) \ , \nonumber \\&(q_h+\bar{q}_{h})^{(n_f+1)}\left( x,m_h^2\right) = A_{\textrm{hq}}^{\textrm{S}}(x)\otimes \Sigma ^{(n_f)}\left( x,m_h^2\right) + A_{\textrm{hg}}^{\textrm{S}}(x)\otimes g^{(n_f)}\left( x,m_h^2\right) \ , \nonumber \\&\Sigma ^{(n_f+1)}\left( x,m_h^2\right) = \Sigma ^{\, (n_f)}\left( x,m_h^2\right) + \left[ A^{\mathrm{NS,+}}_{qq,h}(x) + A^{\textrm{PS}}_{qq,h}(x) + A_{\textrm{hq}}^{\textrm{S}}(x)\right] \otimes \Sigma ^{(n_f)}\left( x,m_h^2\right) \nonumber \\&\qquad \qquad \qquad + \left[ A^{\textrm{S}}_{qg,h}(x) + A_{\textrm{hg}}^{\textrm{S}}(x) \right] \otimes g^{(n_f)}\left( x,m_h^2\right) , \end{aligned}$$

with $q_h=\bar{q}_h$. Up to N$^3$LO the matching coefficients have the following expansions in $\alpha _s$

6 $$\begin{aligned} A^{\mathrm{NS,\pm }}_{qq,h}(x)&= \left( \frac{\alpha _s\left( m_h^2\right) }{2\pi } \right) ^2 A^{\mathrm{NS,\pm ,(2)}}_{qq,h}(x) + \left( \frac{\alpha _s\left( m_h^2\right) }{2\pi } \right) ^3 A^{\mathrm{NS,\pm ,(3)}}_{qq,h}(x) \ , \nonumber \\ A^{\textrm{S}}_{gk,h}(x)&= \left( \frac{\alpha _s\left( m_h^2\right) }{2\pi } \right) ^2 A^{\mathrm{S,(2)}}_{gk,h}(x) + \left( \frac{\alpha _s\left( m_h^2\right) }{2\pi } \right) ^3 A^{\mathrm{S,(3)}}_{gk,h}(x), \quad k=q,g \ , \nonumber \\ A^{\textrm{S}}_{hk}(x)&= \left( \frac{\alpha _s\left( m_h^2\right) }{2\pi } \right) ^2 A^{\mathrm{S,(2)}}_{hk}(x) + \left( \frac{\alpha _s\left( m_h^2\right) }{2\pi } \right) ^3 A^{\mathrm{S,(3)}}_{hk}(x), \quad k=q,g \ , \nonumber \\ A^{\textrm{PS}}_{qq,h}(x)&= \left( \frac{\alpha _s\left( m_h^2\right) }{2\pi } \right) ^3 A^{\mathrm{PS,(3)}}_{qq,h}(x) \, , \nonumber \\ A^{\textrm{S}}_{qg,h}(x)&= \left( \frac{\alpha _s\left( m_h^2\right) }{2\pi } \right) ^3 A^{\mathrm{S,(3)}}_{qg,h}(x)\,. \end{aligned}$$

At $\mathcal {O}(\alpha _S^2)$ we have that $A^{\mathrm{NS,+}}_{qq,h}(x) = A^{\mathrm{NS,-}}_{qq,h}(x)$ whereas they start to differ at $\mathcal {O}(\alpha _S^3)$. The NNLO matching coefficients were computed in [71]^5^ and the N$^3$LO matching coefficients in [72–85].^6^ Notice that the above conditions will lead to small discontinuities of the PDFs in its evolution in $Q^2$, which are cancelled by similar matching terms in the coefficient functions in massive VFN schemes, resulting in continuous physical observables. In particular, the heavy-quark PDFs start from a non-zero value at threshold at NNLO, which sometimes can even be negative.

The corresponding N$^3$LO relation for the matching of the $\overline{\textrm{MS}}$ coupling constant at the heavy quark threshold $m^2_h$ is given by

7 $$\begin{aligned} \alpha _s^{\, (n_f+1)}\left( m_h^2\right) = \alpha _s^{\, (n_f)} \left( m_h^2\right) + C_2 \left( \frac{\alpha _s^{\, (n_f)} \left( m_h^2\right) }{2\pi } \right) ^3 + C_3 \left( \frac{\alpha _s^{\, (n_f)} \left( m_h^2\right) }{2\pi } \right) ^4 \,\,, \end{aligned}$$

where the matching coefficients $C_2$ and $C_3$ were computed in [86, 87]. The value and the form of the matching coefficients in Eqs. (4, 5) depend on the scheme used for the quark masses; by default in hoppet quark masses are taken to be pole masses, though the option exists for the user to supply and have thresholds crossed at $\overline{\textrm{MS}}$ masses, but only up to NNLO. We note that in the current implementation in hoppet the matching can only be performed at the matching point that corresponds to the heavy-quark masses themselves.

Both evolution and threshold matching preserve the momentum sum rule

8 $$\begin{aligned} \int _0^1 dx~x \left( \Sigma \left( x,Q^2\right) +g\left( x,Q^2\right) \right) =1 , \end{aligned}$$

and valence sum rules

9 $$\begin{aligned} \int _0^1 dx\, \left[ q\left( x,Q^2\right) -{{\bar{q}}}\left( x,Q^2\right) \right] = \left\{ \begin{array}{ll} 1, &  \text {for } q = d \text { (in proton)}\\ 2, &  \text {for } q = u \text { (in proton)}\\ 0, &  \text {other flavours} \end{array} \right. \nonumber \\ \end{aligned}$$

as long as they hold at the initial scale (occasionally not the case, e.g. in modified LO sets for Monte Carlo generators [88]).

The default basis for the PDFs, called the human representation in hoppet, is such that the entries in an array pdf(-6:6) of PDFs correspond to:

10 $$\begin{aligned} {\bar{t}}={-6}, \,\,\, {\bar{b}}={-5}, \,\,\, {\bar{c}}={-4}, \,\,\, \nonumber {\bar{s}}= &   {-3}, \,\,\, \nonumber {\bar{u}}={-2}, \,\,\, \nonumber {\bar{d}}={-1}, \,\,\, \\ g= &   {0}, \,\,\, \\ \nonumber d={1}, \,\,\, \nonumber u={2}, \,\,\, \nonumber s={3}, \,\,\, \nonumber c= &   {4}, \,\,\, \nonumber b={5},\,\,\, \nonumber t={6}. \nonumber \end{aligned}$$

However, this representation leads to a complicated form of the evolution equations. The splitting matrix can be simplified considerably (made diagonal except for a $2\times 2$ singlet block) by switching to a different flavour representation, which is named the evln representation, for the PDF set, as explained in detail in [89, 90]. This representation is described in Table 1.

In the evln basis, the gluon evolves coupled to the singlet PDF $\Sigma$, and all non-singlet PDFs evolve independently. Notice that the representations of the PDFs are preserved under linear operations, so in particular they are preserved under DGLAP evolution. The conversion from the human to the evln representations of PDFs requires that the number of active quark flavours $n_f$ be specified by the user, as described in Section 5.1.2 of Ref. [1].

**Table 1:** The evolution representation (called evln in hoppet) of PDFs with $n_f$ active quark flavours in terms of the human representation

i	name	$q_i$
$-6\ldots -(n_f+1)$	$q_i$	$q_i$
$-n_f\ldots -2$	$q_{\textrm{NS},i}^{-}$	$(q_i - {{\bar{q}}}_i) - (q_1 - {{\bar{q}}}_1)$
-1	$q_{\textrm{NS}}^{V}$	$\sum _{j=1}^{n_f} (q_j - {{\bar{q}}}_j)$
0	g	gluon
1	$\Sigma$	$\sum _{j=1}^{n_f} (q_j + {{\bar{q}}}_j)$
$2\ldots n_f$	$q_{\textrm{NS},i}^{+}$	$(q_i + {{\bar{q}}}_i) - (q_1 + {{\bar{q}}}_1)$
$(n_f+1)\ldots 6$	$q_i$	$q_i$

In hoppet, unpolarised DGLAP evolution is available up to N$^3$LO in the $\overline{\textrm{MS}}$ scheme, while for the DIS scheme only evolution up to NLO is available, but without the NLO heavy-quark threshold matching conditions. For polarised evolution up to NLO only the $\overline{\textrm{MS}}$ scheme is available. The variable factscheme takes different values for each factorisation scheme:

**Table:** 

factscheme	Evolution
1	unpolarised $\overline{\textrm{MS}}$ scheme
2	unpolarised DIS scheme
3	polarised $\overline{\textrm{MS}}$ scheme

Note that mass thresholds are currently missing in the DIS scheme.

The extension to QED is conceptually straightforward. Further discussion of that is given in Sect. 6.

## Brief summary of hoppet structure

hoppet works in *x*-space. It represents PDFs and splitting functions on grids, typically multiple nested grids, each uniform in $y= \ln 1/x$, with the nesting involving smaller spacings and smaller *y* ranges so as to achieve good accuracy not just at small *x* but also large *x*. The underlying convolutions of splitting functions with PDFs effectively use piecewise polynomial interpolations of the PDFs. The convolutions of the splitting functions with individual basis polynomials are pre-evaluated using adaptive Gaussian integration. Evolution equations are solved using Runge–Kutta methods.

**Table 2:** Core methods of the streamlined interface in Fortran and C**++**. In Python, hoppetStart(...) is to be replaced with hoppet.Start(...), and routines like hoppet.Eval(...) and LHAsub(x,Q) return f rather than taking f as an argument and filling it

**Configuration (optional)**	
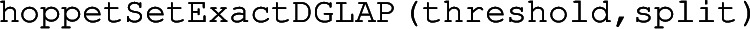	Sets use of exact NNLO mass thresholds and splitting functions (default: both false).
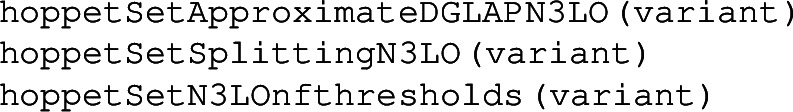	Sets variants for the choice of N$^3$LO splitting functions and thresholds, cf. Sec. 4.
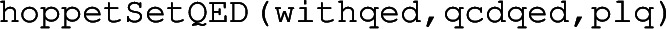	Sets QED evolution and its options (cf. Sec. 6; default: all false).
**Initialisation**	
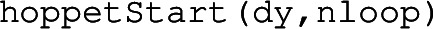	Sets up a compound grid with spacing in $\ln 1/x$ of dy at small *x*, extending to $y = 12$ and numerical order $\texttt {=}-6$. The *Q* range for the tabulation will be $1\;\textrm{GeV}< Q<28 \;\textrm{TeV}$, dlnlnQ=dy/4 and the factorisation scheme is ${\overline{\textrm{MS}}}$ (factscheme_MSbar).
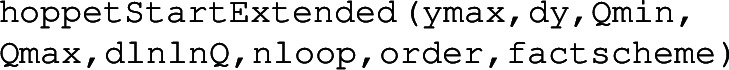	More general initialisation.
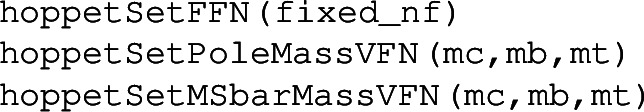	Set heavy flavour scheme ($\overline{\textrm{MS}}$ available only to NNLO).
**Normal evolution**	
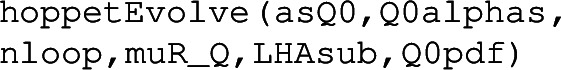	PDF evolution: specifies the coupling asQ0 at a scale Q0alphas, the number of loops for evol., nloop, the ratio (muR_Q) of ren. to fact. scales, the name of a subroutine LHAsub(x,Q,f) that fills f(-6:6), and the scale Q0pdf at which one starts the PDF evolution. Note: LHAsub is only called at scale Q0pdf and in C**++** f[iflv] spans iflv=0..12.
**Cached evolution**	
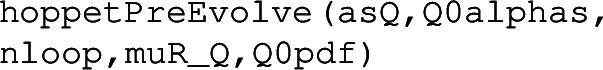	Preparation of a cached evolution.
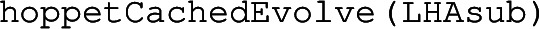	Perform cached evolution with the initial condition at Q0pdf from a routine LHAsub with LHAPDF-like interface. Note: LHAsub only called at scale Q0pdf.
**Evaluation**	
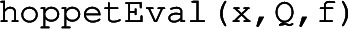	On return, f(-6:6) contains all flavours of the PDF set(multiplied by *x*). In C**++**, the array indices span 0 to 12. Increase upper bound by 5 with QED.
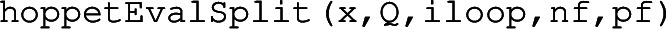	On return, pf(-6:6) contains the (cached) convolution of the iloop splitting function ($1=\text {LO}$) with the tabulated PDF for the given nf. One can chain splitting functions up to ${{\mathcal {O}}}\left( \alpha _s^4\right)$, e.g. iloop=31 gives $P_\text {NNLO}\otimes P_\text {LO} \otimes f$.
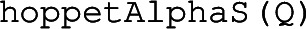	Returns the coupling at scale *Q*.
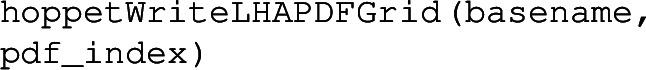	Write an LHAPDF grid file to basename_nnnn.dat where nnnn is pdf_index; if pdf_index is 0, also write a template basename.info file.
**Cleanup (optional)**	
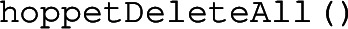	Deletes all storage allocated by the streamlined interface

The code has two interfaces. For simple usage, it provides a so-called “streamlined” interface, giving high-level access to the functionality that is most widely needed. It is available from Fortran, C**++**and, as of v2, Python (cf. Sec. 7). Its main routines are listed in Table 2. The functionality includes evolving to fill a PDF tabulation and then accessing that PDF tabulation at given *x*, *Q* points. For faster tabulation of many distinct initial conditions, one can pre-determine (cache) the evolution operators between the different *Q* scales at which the PDF is tabulated, and then repeatedly apply that cached evolution. The streamlined interface also provides access to convolutions of the various orders of splitting functions with the tabulated PDF.

**Table 3:** Core objects in the general interface

	$x-$space grid definition
	Holds a ‘grid quantity’ (e.g. gluon PDF)
	Grid representation of a (13-flavour) PDF set
	Convolution operator (*i.e.* splitting function)
	Splitting matrix (with full flavour structure)
	Heavy quark mass-threshold matrix
	DGLAP holder (i.e. all splitting and mass-threshold matrices)
	Running coupling
	Evolution operator (linked list of split & mass-threshold matrices)
	PDF set tabulated in *x* & *Q*

For more advanced usage there is a “general” interface (sometimes called the object-oriented interface, though it is only partially so). It gives access to the various low-level objects that are useful in DGLAP evolution, such as splitting functions, splitting matrices, tabulations of PDFs, etc. The main objects are listed in Table 3. Up to v2.1, that interface is accessible only in modern Fortran, though in due course we expect to extend it to other languages. We refer the reader to the original manual [1] for an extended discussion of the general interface. For some uses, it can be convenient to initialise hoppet with the streamlined interface and then access the underlying objects in Fortran from the streamlined_interface module.

Various examples are available with the two sets of interfaces, to be found in the https://github.com/hoppet-code/hoppet/blob/hoppet-2.1.1/examples/ directory of the repository. This document will focus mostly on the streamlined interface, though in a places we will also discuss key additions to the general interface.

## QCD evolution at N$^3$LO

In recent years significant progress in determining the perturbative components needed for unpolarised QCD evolution at N$^3$LO has been made (cf. 2 for technical details on the evolution at N$^3$LO). This has now reached the stage that two PDF groups have released fits at approximate N$^3$LO (aN$^3$LO) accuracy [49, 50] along with their combination in Ref. [91]. Of the three contributions that are needed at full N$^3$LO accuracy only two are fully known. In particular the four-loop $\beta$-function [92, 93] and associated mass thresholds [86] have been known for a very long time. On the other hand the intricate calculations of the three-loop matching relations needed for both the single and two mass VFNS were only very recently completed [72–81, 83, 84, 94]. Finally the four-loop splitting functions entering the DGLAP equation are currently not known exactly, except for certain $n_f$-dependent terms [43–48, 95]. However, enough Mellin-moments have been computed that together with the exact pieces just mentioned and known small- and large-*x* behaviours, approximate splitting functions suitable for phenomenology can be reliably determined [49–56]. We have therefore incorporated the aforementioned pieces, using code that is publicly available with those references, with the intention of updating the splitting functions as they become more precisely known.

### Interface

For the specific implementation in hoppet version 2.0.0 we rely on the approximations computed in Refs. [44, 45, 52–56] (FHMPRUVV), the implementation of the three-loop (single-mass) VFNS coefficients as found in Ref. [85], which contains code associated with Refs. [82, 84] and our own implementation of the four-loop running coupling.^7^ Since the implementation here extends core features of hoppet that were already available in version 1.1.0, very little is needed on the side of the user to invoke the evolution. Most importantly all routines that take an nloop argument, e.g. hoppetStart, and hoppetEvolve (or InitDglapHolder, InitRunningCoupling in the general interface) now support nloop = 4. The user also has a few choices they can make in terms of the splitting functions and mass thresholds used.

Firstly, there have been successively improved approximations to the splitting functions. The user can control which series of approximation to use by making a call to the subroutine hoppetSetApproximateDGLAPN3LO (splitting_approx). At the time of writing, three options are available for the splitting_approx:

- n3lo_splitting_approximation_up_to_2310_05744, with the approximations in papers up to and including Ref. [54];
- n3lo_splitting_approximation_up_to_2404_09701, with the approximations in papers up to and including Ref. [55];
- n3lo_splitting_approximation_up_to_2410_08089, the default value at the time of writing, with the approximations in papers up to and including Ref. [56].

These constants, and the others discussed in this section, are defined in the dglap_choices module in Fortran. In C**++**they are in the hoppet namespace, as defined in https://github.com/hoppet-code/hoppet/blob/hoppet-2.1.1/src/hoppet.h.

The approximate splitting functions come with an uncertainty band. The user has a choice between the two extremities of the band and an average of those extremities. They can make the choice by calling hoppetSetSplittingN3LO(variant), where variant is one of

- n3lo_splitting_Nfitav: the average (the default),
- n3lo_splitting_Nfiterr1: one of the two extremities,
- n3lo_splitting_Nfiterr2: the other of the two. extremities.

Besides the splitting functions, we have also incorporated the single-mass thresholds up to N$^3$LO, as calculated in Refs. [72–81, 83, 84]. As of version 2.1.0, the N$^3$LO thresholds are available in two forms, using code from Refs. [82, 85]. One can choose which form to use by calling hoppetSetN3LOnfthresholds(n3lo_threshold_choice), with one of the following values for n3lo_threshold_choice:

- n3lo_nfthreshold_libOME: this uses piecewise high-order Laurent-series expansions in different regions of *x*, with a stated accuracy of at least $2048\epsilon$ where $\epsilon$ is the precision of a 64-bit real variable. It relies on the libome C**++**library from https://gitlab.com/libome/libome. Initialisation time is below $1\,\text {s}$. This is the default choice.
- n3lo_nfthreshold_exact_fortran: all contributions are exact, except for the $A_\text {hg}^\text {S}$ term in Eq. (5), which is based on piecewise expansions. The exact contributions make extensive use of hplog5 [96] calls and lead to an initialisation time of $10{-}30\,\text {s}$. The piecewise expansions for $A_\text {hg}^\text {S}$ are less accurate than with the n3lo_nfthreshold_libOME choice.

### Reference results

In Table 4 we show the results of the full N$^3$LO evolution in the VFNS, using the most up-to-date perturbative input at the time of writing this release note. To assess the evolution we take the initial condition of Ref. [8] at an initial scale $\sqrt{2}~\text {GeV}$ and $n_f=3$. We evolve to $Q=100~\text {GeV}$. The numbers are obtained using parametrised NNLO splitting functions, but exact mass thresholds at this order. The table has been generated with $\texttt {dy}=0.05$, $\texttt {dlnlnQ}=\text {dy/4}$. Increasing $\texttt {dy}=0.10$ leaves the results unchanged at the precision shown, while going to $\texttt {dy}=0.20$ for higher speed would change the results by a relative amount below $10^{-4}$.

Table 4 cannot be directly compared to the benchmarking tables of Ref. [97] because they do not include the mass thresholds in the N$^3$LO evolution. To facilitate comparisons we therefore additionally provide Table 5, corresponding to fixed-flavour ($n_f=4$) evolution, with the choice of n3lo_splitting_approximation_up_to_2310_05744. Of the MSHT and NNPDF results, the NNPDF results (Table 2 of Ref. [97]) are closer to ours.

The results in both tables can be regenerated with the help of the following script, which uses the Python interface of Sect. 7: https://github.com/hoppet-code/hoppet/blob/hoppet-2.1.1/src/benchmarking/tabulation_crosscheck_2406_16188.py.

**Table 4:** N$^3$LO evolution of the initial condition given in Section 4.4 of Ref. [8], using the same notation where $a\cdot 10^{b} = a^b$. The evolution is performed taking the initial condition at $\sqrt{2}~\text {GeV}$ (just below the charm mass) and evolving in the VFNS up to $Q=100~\text {GeV}$, with a charm-quark pole mass of $1.414213563\;\textrm{GeV}$ and a bottom-quark pole mass of $4.5\;\textrm{GeV}$. The NNLO splitting functions are the parametrised form (nnlo_splitting_variant = nnlo_splitting_param), to facilitate comparisons by other groups, and the N$^3$LO splitting functions use the n3lo_splitting_approximation = n3lo_splitting_approximation_up_to_2410_08089 choice

*x*	$u-{\bar{u}}$	$d-{\bar{d}}$	${\bar{d}}-{\bar{u}}$	$2({\bar{u}}+{\bar{d}})$	$s+{\bar{s}}$	$c+{\bar{c}}$	$b+{\bar{b}}$	*g*
$10^{-7}$	$1.0589^{-4}$	$4.8664^{-5}$	$8.1967^{-6}$	$1.6234^{+2}$	$8.0100^{+1}$	$7.7207^{+1}$	$6.5254^{+1}$	$1.1238^{+3}$
$10^{-6}$	$5.9691^{-4}$	$3.2634^{-4}$	$3.3146^{-5}$	$7.6786^{+1}$	$3.7544^{+1}$	$3.5836^{+1}$	$2.9889^{+1}$	$5.1159^{+2}$
$10^{-5}$	$3.0235^{-3}$	$1.7532^{-3}$	$1.3081^{-4}$	$3.5436^{+1}$	$1.7044^{+1}$	$1.6106^{+1}$	$1.3142^{+1}$	$2.2224^{+2}$
$10^{-4}$	$1.4079^{-2}$	$8.2354^{-3}$	$4.9511^{-4}$	$1.5611^{+1}$	$7.2731^{+0}$	$6.7862^{+0}$	$5.3294^{+0}$	$8.8594^{+1}$
$10^{-3}$	$6.0849^{-2}$	$3.5086^{-2}$	$1.7751^{-3}$	$6.3823^{+0}$	$2.7798^{+0}$	$2.5204^{+0}$	$1.8516^{+0}$	$3.0349^{+1}$
$10^{-2}$	$2.3361^{-1}$	$1.3074^{-1}$	$5.8324^{-3}$	$2.2673^{+0}$	$8.5415^{-1}$	$7.0444^{-1}$	$4.6228^{-1}$	$7.7859^{+0}$
0.1	$5.4846^{-1}$	$2.6950^{-1}$	$9.9965^{-3}$	$3.8453^{-1}$	$1.1248^{-1}$	$6.8296^{-2}$	$3.7899^{-2}$	$8.4964^{-1}$
0.3	$3.4441^{-1}$	$1.2761^{-1}$	$2.9457^{-3}$	$3.4575^{-2}$	$8.8873^{-3}$	$3.9659^{-3}$	$2.0846^{-3}$	$7.8697^{-2}$
0.5	$1.1790^{-1}$	$3.0597^{-2}$	$3.6526^{-4}$	$2.3206^{-3}$	$5.6808^{-4}$	$2.0185^{-4}$	$1.1382^{-4}$	$7.6337^{-3}$
0.7	$1.9329^{-2}$	$2.9648^{-3}$	$1.2848^{-5}$	$5.2429^{-5}$	$1.2662^{-5}$	$3.4020^{-6}$	$2.4956^{-6}$	$3.7094^{-4}$
0.9	$3.3153^{-4}$	$1.6737^{-5}$	$8.0961^{-9}$	$2.5214^{-8}$	$6.6432^{-9}$	$7.6153^{-10}$	$1.4323^{-9}$	$1.1716^{-6}$

**Table 5:** N$^3$LO evolution of the initial condition given in Section 4.4 of Ref. [8], using the same notation where $a\cdot 10^{b} = a^b$. The evolution is performed taking the initial condition at $\sqrt{2}~\text {GeV}$ (just below the charm mass) and evolving in the FFN scheme ($n_f = 4$) up to $Q=100~\text {GeV}$. The NNLO splitting functions are the parametrised form (nnlo_splitting_variant = nnlo_splitting_param), to facilitate comparisons by other groups, and the N$^3$LO splitting functions use the n3lo_splitting_approximation = n3lo_splitting_approximation_up_to_2310_05744 choice. This table can be directly compared to tables 1 and 2 in Ref. [97]

*x*	$u-{\bar{u}}$	$d-{\bar{d}}$	${\bar{d}}-{\bar{u}}$	$2({\bar{u}}+{\bar{d}})$	$s-{\bar{s}}$	$s+{\bar{s}}$	$c+{\bar{c}}$	*g*
$10^{-7}$	$9.8370^{-5}$	$4.5171^{-5}$	$7.5013^{-6}$	$1.4888^{+2}$	$-2.9105^{-5}$	$7.3368^{+1}$	$7.2653^{+1}$	$1.0851^{+3}$
$10^{-6}$	$5.6405^{-4}$	$3.0895^{-4}$	$3.0730^{-5}$	$7.1925^{+1}$	$-4.6739^{-5}$	$3.5111^{+1}$	$3.4544^{+1}$	$5.0392^{+2}$
$10^{-5}$	$2.8946^{-3}$	$1.6810^{-3}$	$1.2302^{-4}$	$3.3868^{+1}$	$-3.5766^{-6}$	$1.6258^{+1}$	$1.5808^{+1}$	$2.2292^{+2}$
$10^{-4}$	$1.3633^{-2}$	$7.9832^{-3}$	$4.7274^{-4}$	$1.5188^{+1}$	$\phantom {-}2.1123^{-4}$	$7.0599^{+0}$	$6.7033^{+0}$	$9.0268^{+1}$
$10^{-3}$	$5.9567^{-2}$	$3.4382^{-2}$	$1.7232^{-3}$	$6.3028^{+0}$	$\phantom {-}3.9314^{-4}$	$2.7387^{+0}$	$2.4621^{+0}$	$3.1350^{+1}$
$10^{-2}$	$2.3130^{-1}$	$1.2962^{-1}$	$5.7645^{-3}$	$2.2675^{+0}$	$-1.9644^{-4}$	$8.5255^{-1}$	$6.6402^{-1}$	$8.1568^{+0}$
0.1	$5.5131^{-1}$	$2.7140^{-1}$	$1.0085^{-2}$	$3.8980^{-1}$	$-3.1812^{-4}$	$1.1388^{-1}$	$5.9843^{-2}$	$9.0615^{-1}$
0.3	$3.5044^{-1}$	$1.3015^{-1}$	$3.0145^{-3}$	$3.5426^{-2}$	$-3.8409^{-5}$	$9.0900^{-3}$	$3.3507^{-3}$	$8.4431^{-2}$
0.5	$1.2112^{-1}$	$3.1518^{-2}$	$3.7779^{-4}$	$2.3973^{-3}$	$-3.3053^{-6}$	$5.8501^{-4}$	$1.7709^{-4}$	$8.1568^{-3}$
0.7	$2.0078^{-2}$	$3.0889^{-3}$	$1.3448^{-5}$	$5.4598^{-5}$	$-1.1810^{-8}$	$1.3105^{-5}$	$3.6963^{-6}$	$3.9248^{-4}$
0.9	$3.5128^{-4}$	$1.7793^{-5}$	$8.6472^{-9}$	$2.6378^{-8}$	$-1.5848^{-10}$	$6.8222^{-9}$	$2.6776^{-9}$	$1.2262^{-6}$

**Fig. 1:**
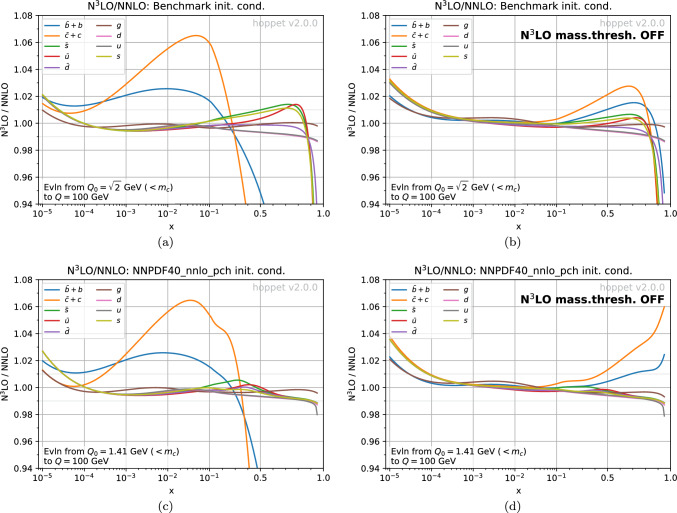
Ratio of the PDFs at $100\;\textrm{GeV}$ with N$^3$LO evolution versus NNLO evolution. The evolution starts from the same initial condition at NNLO and N$^3$LO, at an initial scale $Q_0\simeq 1.41\;\textrm{GeV}$. In the upper plots, we use the standard benchmark initial condition. In the lower plots, the initial condition is the NNPDF40_pch_nnlo_as_01180 PDF [98] at $Q_0$. The left-hand plots show the ratio with N$^3$LO evolution including N$^3$LO mass thresholds, while the right-hand plots show the evolution without the N$^3$LO mass thresholds. In all cases, the N$^3$LO evolution uses n3lo_splitting_approximation_up_to_2410_08089

We close this section by illustrating the impact of N$^3$LO versus NNLO evolution, Fig. 1. Let us first focus on the top-left plot, Fig. 1a. We start with the benchmark initial condition at the standard low scale of $Q_0 = \sqrt{2}\;\textrm{GeV}$, just below a charm mass of $m_c = 1.414213563 \;\textrm{GeV}$, so as to ensure that the evolution starts with $n_f = 3$. We then evolve the PDF separately with N$^3$LO and NNLO evolution, and show the N$^3$LO/NNLO ratio at $Q = 100\;\textrm{GeV}$. Each line corresponds to a different flavour. For light flavours, in the range that is relevant to the LHC at central rapidities, $10^{-4} \lesssim x \lesssim 0.5$, the effect of N$^3$LO corrections on the evolution of the light-flavour PDFs is generally below a percent, and typically less than or around half a percent. For heavy flavour, the effect is much more significant, with a $\sim 6\%$ effect on the charm distribution for $0.01 \lesssim x \lesssim 0.1$ and about $-15\%$ at $x=0.5$.

Figure 1b is the analogous plot with the N$^3$LO mass-threshold contributions turned off. It illustrates that the large effects on the charm and bottom PDFs are a consequence mainly of N$^3$LO mass-thresholds, not the N$^3$LO splitting functions. Comparing Figs. 1a and b for light flavours, one sees that the $0.5\%$ effects are coming both from the N$^3$LO splitting functions and the N$^3$LO mass thresholds.

Finally, Fig. 1c and d show analogous plots with an initial condition taken from the NNPDF40_pch_nnlo_as_01180 PDF set [98] at a similar $Q_0 = 1.41 \;\textrm{GeV}$ (again below the charm threshold, $m_c = 1.51\;\textrm{GeV}$). The results are broadly similar, showing that our conclusions about the size of N$^3$LO effects are robust with respect to the choice of PDF.

## Hadronic structure functions

As of hoppet version 2.0.0, the code provides access to the massless hadronic structure functions. The structure functions are expressed as convolutions of a set of massless hard coefficient functions and PDFs, and make use of the tabulated PDFs and streamlined interface. They are provided such that they can be used directly for cross section computations in DIS or VBF, as implemented for example in disorder [34] and the proVBFH package [30–33, 99, 100].

The massless structure functions have been found to be in good agreement with those that can be obtained with APFEL**++** [2, 3] (at the level of $10^{-5}$ relative precision). The benchmarks with APFEL**++** and the code used to carry them out are described in detail in Ref. [9] and at https://github.com/alexanderkarlberg/n3lo-structure-function-benchmarks. A simplified version of that benchmark is also included in the hoppet repository and can be found in https://github.com/hoppet-code/hoppet/blob/hoppet-2.1.1/benchmarking/structure_functions_benchmark_checks.f90. Technical details on the implementation of the structure functions in hoppet can be found in Refs. [9, 101, 102], and here we mainly focus on the code interface.

The structure functions have been implemented including only QCD corrections up to N$^3$LO using both the exact and parametrised coefficient functions found in Refs. [89, 90, 103–107],^8^ and can make use of PDFs evolved at N$^3$LO as described in Sect. 4. The structure functions can also be computed using PDFs interfaced through LHAPDF [108].

### Initialisation

The structure functions can be accessed by using the structure_functions module. They can also be accessed through the streamlined interface by prefixing hoppet, as described later in Sect. 5.3. The description here corresponds to an intermediate-level interface, which relies on elements such as the grid and splitting functions having been initialised in the streamlined interface, through a call to hoppetStart or hoppetStartExtended, cf. Section 8 of Ref. [1].^9^ After this initialisation has been carried out, one calls 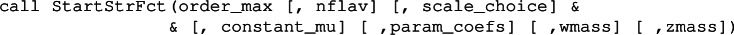


specifying as a minimum the perturbative order — currently order_max $$ \le 4$$ (order_max $$ =1$$ corresponds to LO).

If nflav is not passed as an argument, the structure functions are initialised to support a variable flavour-number scheme (the masses that are used at any given stage will be those set in the streamlined interface). Otherwise a fixed number of light flavours is used, as indicated by nflav, which speeds up initialisation. Note that specifying a variable flavour-number scheme only has an impact on the evolution and on $n_f$ terms in the coefficient functions. The latter, however always assume massless quarks. Hence in both the fixed and variable flavour-number scheme the structure functions should not be considered phenomenologically reliable if *Q* is comparable to the quark mass. Together xR, xF, scale_choice, and constant_mu control the renormalisation and factorisation scales and the degree of flexibility that will be available in choosing them at later stages. Specifically the (integer) scale_choice argument should be one of the following values (defined in the structure_functions module):

- scale_choice_Q (default) means that the code will always use *Q* multiplied by xR or xF as the renormalisation and factorisation scale respectively (with xR or xF as set at initialisation).
- scale_choice_fixed corresponds to a fixed scale constant_mu, multiplied by xR or xF as set at initialisation.
- scale_choice_arbitrary allows the user to choose arbitrary scales at the moment of evaluating the structure functions. In this last case, the structure functions are saved as separate arrays, one for each perturbative order, and with dedicated additional arrays for terms proportional to logarithms of $Q/\mu _F$. This makes for a slower evaluation compared to the two other scale choices.

If param_coefs is set to .true. (its default) then the structure functions are computed using the NNLO and N$^3$LO parametrisations found in Refs. [89, 90, 103–106], which are stated to have a relative precision of a few permille (order by order) except at particularly small or large values of *x*. Alternatively, it is possible to ask for the exact coefficient functions.^10^ Since the expressions are large, and slow down the compilation, one must explicitly request their compilation with the-DHOPPET_USE_EXACT_COEF=ON CMake flag. Having done that, one then has the option of setting param_coefs to .false.. This will lead the initialisation to become quite slow (up to two minutes rather than a few seconds). Given the good accuracy of the parametrised coefficient functions, they are to be preferred for most applications.

The masses of the electroweak vector bosons are used only to calculate the weak mixing angle, $\sin ^2 \theta _W = 1 - (m_W/m_Z)^2$, which enters in the neutral-current structure functions.

At this point all the tables that are needed for the structure functions have been allocated. In order to fill the tables, one first needs to set up the running coupling and evolve the initial PDF with hoppetEvolve, as described in Section 8.2 of Ref. [1].

With the PDF table filled in the streamlined interface one calls 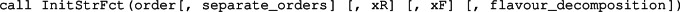


specifying the order at which one would like to compute the structure functions. The logical flag separate_orders should be set to .true. if one wants access to the individual coefficients of the perturbative expansion as well as the sum up to some maximum order, order. With scale_choice_Q and scale_choice_fixed, the default of .false. causes only the sum over perturbative orders to be stored. This gives faster evaluations of structure functions because it is only necessary to interpolate the sum over orders, rather than interpolate one table for each order. With scale_choice_arbitrary, the default is .true., which is the only allowed option, because separate tables for each order are required for the underlying calculations.

Finally, the optional flag flavour_decomposition controls an experimental feature of giving access to the structure functions decomposed into their underlying quark flavours without the associated vector boson couplings. It is currently only possible to access the structure functions in this way up to NLO, and since the feature is not fully mature we invite interested readers to inspect the source code directly for more information.

### Accessing the structure functions

At this point the structure functions can be accessed as in the following example 
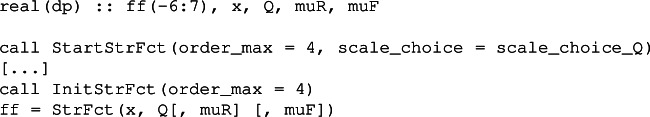


at the value *x* and *Q*. With scale_choice_arbitrary, the muR and muF arguments must be provided. With other scale choices, they do not need to be provided, but if they are then they should be consistent with the original scale choice. The structure functions in this example are stored in the array ff. The components of this array can be accessed through the indices
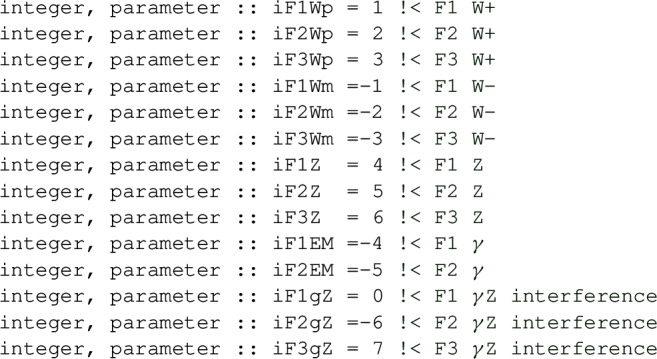


For instance one would access the electromagnetic $F_1$ structure function through ff(iF1EM). It is returned at the order_max that was specified in InitStrFct. The structure functions can also be accessed order by order if the separate_orders flag was set to .true. when initialising. They are then obtained as follows
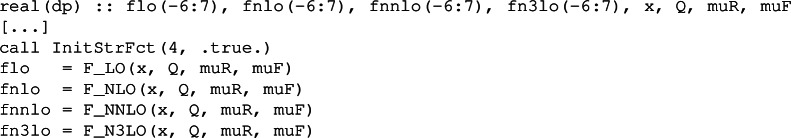


The functions return the individual contributions at each order in $\alpha _s$, including the relevant factor of $\alpha _s^n$. Hence the sum of flo, fnlo, fnnlo, and fn3lo would be equal to the full structure function at N$^3$LO as contained in ff in the example above. Note that in the F_LO etc. calls, the muR and muF arguments are not optional and that when a prior scale choice has been made (e.g. scale_choice_Q) they are required to be consistent with that prior scale choice.

An example of structure function evaluations using the Fortran 90 interface is to be found in examples/f90/structure_functions_example.f90.

### Streamlined interface

The structure functions can also be accessed through the streamlined interface, so that they may be called for instance from C/C**++**. The functions to be called are very similar to those described above. For simple usage one can call 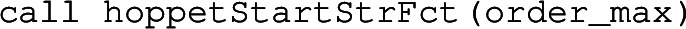


where order_max-1 is the maximal power of $\alpha _s$. Alternatively, the extended version of the interface, hoppetStartStrFctExtended, takes all the same arguments as StartStrFct described above. One difference is that in order to use a variable flavour scheme the user should set nflav to a negative value. After evolving or reading in a PDF, the user then calls 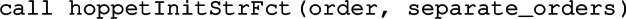


to initialise the actual structure functions. The structure functions can then be accessed through the subroutines
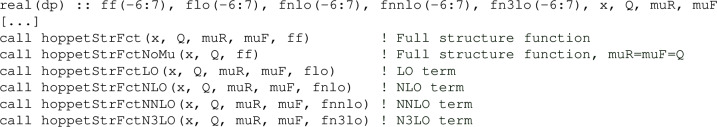


The C**++** header contains indices for the structure functions and scale choices, which are all in the hoppet namespace. 
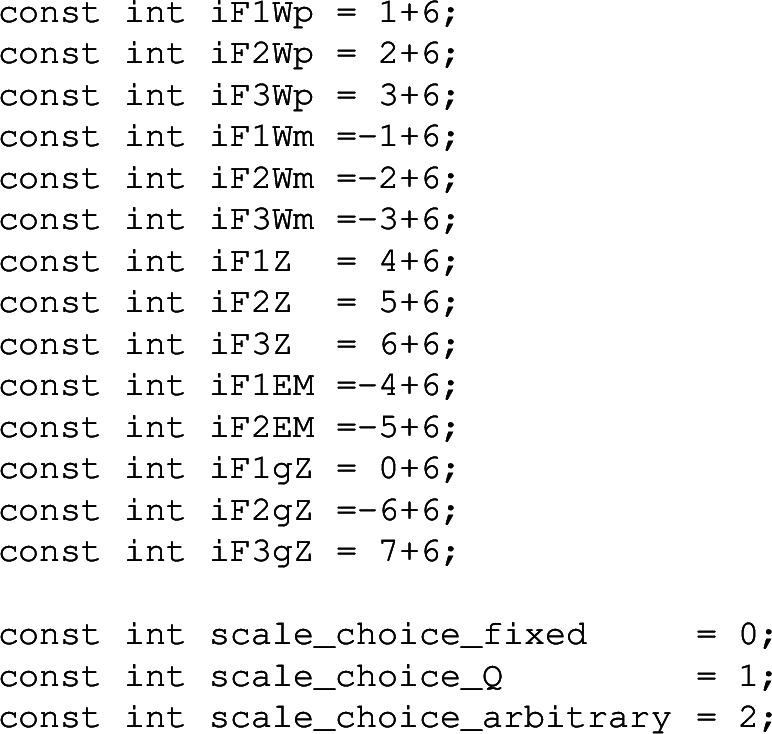


Note that in C**++** the structure function indices start from 0 and that the C**++** array that is to be passed to functions such as hoppetStrFct would be defined as double ff[14].

An example of structure function evaluations using the C**++** version of the streamlined interface is to be found in examples/cpp/structure_functions_example.cc and a Python example is similarly to be found at https://github.com/hoppet-code/hoppet/blob/hoppet-2.1.1/examples/python/structure_function_example.py.

**Fig. 2:**
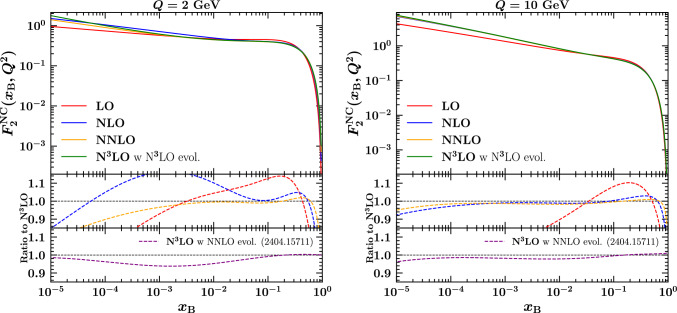
The structure function $F_2^{\textrm{NC}}$ plotted as a function of $x_{\textrm{B}}$ in the range $[10^{-5}:0.9]$ at $Q=2$ GeV (left) and $Q=10$ GeV (right). Each plot displays the curves at LO, NLO, NNLO, and N$^3$LO with the middle panel showing the ratio to N$^3$LO. The lowest panel shows the ratio of the N$^3$LO with NNLO evolution to the full N$^3$LO result. Adapted from Ref. [9] using the full N$^3$LO evolution

Finally we present a small update on the results presented in Ref. [9]. That reference was published before the full N$^3$LO evolution had been implemented in hoppet, and the N$^3$LO structure functions were therefore obtained with NNLO evolution. A full update of the tables and plots is beyond the scope of the current work, but for illustrative purposes we present here an updated version of Fig. 3 of Ref. [9]. The update uses the full N$^3$LO evolution and is shown as Fig. 2.

It shows the full charged-lepton neutral current $F_2$ structure function ($F_2^{\textrm{NC}}$) at various perturbative orders, for two values of *Q* (2 GeV and 10 GeV). It also shows the relative difference of the various orders with respect to N$^3$LO, and in the lower panel the ratio of the N$^3$LO result of Ref. [9] to that obtained here with the full evolution. As can be seen by comparing to the original figures and from the lowest panel, the N$^3$LO evolution has the effect at low *Q* of increasing the N$^3$LO $F_2^{\textrm{NC}}$ by a few percent, at least for *x*-values in the range $10^{-4}$ to $10^{-1}$.

## Evolution including QED contributions

The combined QED + QCD evolution, as implemented in hoppet since version 2.0.0 (and earlier in a dedicated qed branch), was first described in Refs. [35–38]. The determination of which contributions to include follows a consistent approach based on the so-called “phenomenological” counting scheme. Within this scheme, one considers the QED coupling $\alpha$ to be of order $\alpha _s^2$, and takes the photon (lepton) PDF to be of order $\alpha L$ ($\alpha ^2 L^2$), where *L* is the logarithm of the ratio of the factorisation scale to a typical hadronic scale and is considered to be of order $L \sim 1/\alpha _s$. In contrast, quark and gluon PDFs are considered to be of order $(\alpha _sL)^n={{\mathcal {O}}}(1)$.^11^ From this point of view, NNLO (3-loop) QCD evolution provides control of terms of order up to $\alpha _s^{n+2} L^n \sim \alpha _s^2$. To achieve a corresponding accuracy when including QED contributions, hoppet has been extended to account for

1. 1-loop QED splitting functions [109], which first contribute at order $\alpha L \sim \alpha _s$, i.e. count as NLO QCD corrections;
2. 1-loop QED running coupling, including lepton and quark thresholds, which first contributes at order $\alpha ^2\,L^2 \sim \alpha _s^2$, i.e. like NNLO QCD;
3. 2-loop mixed QCD-QED splitting functions [110], which first contribute at order $\alpha \alpha _sL \sim \alpha _s^2$, i.e. count as NNLO QCD corrections;
4. optionally, the 2-loop pure QED $P_{\ell q}$ splitting function [111], which brings absolute accuracy $\alpha ^2 L\sim \alpha _s^3$ to the lepton distribution (which starts at $\alpha ^2 L^2 \sim \alpha _s^2$).^12^ In an absolute counting of accuracy, this is not needed. However, if one wants lepton distributions to have the same relative NLO accuracy as the photon distribution, it should be included.

The code could be extended systematically to aim at a higher accuracy. For instance, if one wished to reach N$^3$LO accuracy in the phenomenological counting, one would need to include 3-loop mixed QCD-QED splitting functions at order $\alpha \alpha _s^2$, which contribute at order $\alpha \alpha _s^2 L \sim \alpha _s^3$ but are currently not available, the full 2-loop pure QED splitting functions [111], and the 2-loop mixed QED-QCD contributions to the running of the QED coupling, which contribute at order $\alpha ^2 \alpha _sL^2 \sim \alpha _s^3$ (see e.g. [112]).

The rest of this section is structured as follows: Sect. 6.1 shows how to get QED evolution in the streamlined interface, which is relatively straightforward. Section 6.2 then gives a technical discussion of the implementation of the QED evolution, including details, for example, regarding the choice of QED coupling. Some readers may prefer to skip or skim this on a first reading.

### Streamlined interface with QED effects

The streamlined interface including QED effects works as in the case of pure QCD evolution. One has to add the following call 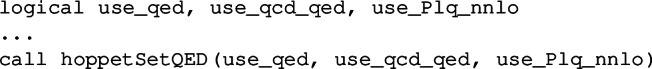


before using the streamlined interface routines. The use_qed argument turns QED evolution on/off at order ${{\mathcal {O}}}(\alpha )$ (i.e. items 1 and 2 in the enumerated list at the beginning of Sec. 6). The use_qcd_qed one turns mixed QCD$\times$QED effects on/off in the evolution (i.e. item 3) and use_Plq_nnlo turns the order $\alpha ^2 P_{\ell q}$ splitting function on/off (i.e. item 4). Without this call, all QED corrections are off.

With the above, the streamlined interface can then be used as normal. E.g. by calling the hoppetEvolve(...) function to fill the PDF table and hoppetEval(x,Q,f) to evaluate the PDF at a given *x* and *Q*. Note that the f array in the latter call must be suitably large, e.g. f(-6:11) for a PDF with leptons (numbering is shifted by $+6$ in C**++**). Examples of the streamlined interface being used with QED evolution can be found in

https://github.com/hoppet-code/hoppet/blob/hoppet-2.1.1/examples/f90/tabulation_example_qed_streamlined.f90

https://github.com/hoppet-code/hoppet/blob/hoppet-2.1.1/examples/cpp/tabulation_example_qed.cc

### Implementation of the QED extension

*QED coupling*

A first ingredient is the setup of the QED coupling object, defined in module qed_coupling: 
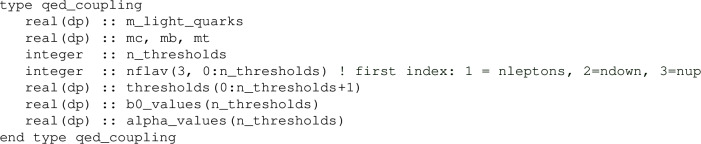


This is initialized through a call to 
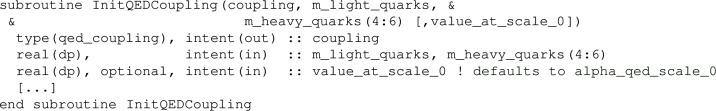


It initialises the parameters relevant to the QED coupling and its running. The electromagnetic coupling at scale zero is set by default to its PDG Thomson value [113] value, unless the optional argument value_at_scale_0 is provided, in which case the latter is taken.

The running is performed at leading order level, using seven thresholds: a common effective mass for the three light quarks (m_light_quarks), the three lepton masses (hard-coded to their 2025 PDG values [114] in the src/qed_coupling.f90 file), and the three masses of the heavy quarks (m_heavy_quarks(4:6)). The common value of the light quark masses is used to mimic the physical evolution in the region $0.1\;\textrm{GeV}\lesssim \mu \lesssim 1\;\textrm{GeV}$, which involves hadronic states. Using a value of $0.1055\;\textrm{GeV}$ generates QED coupling values at the masses of the $\tau$ lepton (1/133.444) and *Z*-boson (1/127.938) that agree to within relative $\sim 1\times 10^{-4}$ (and $1\sigma$) accuracy with the $\overline{\textrm{MS}}$ values ($1/(133.450 \pm 0.008)$ and $1/(127.930 \pm 0.006)$) from the “Electroweak and constraints on New Physics” section of the 2024 Particle Data Group review [114].

The quark and lepton masses are used to set all thresholds where the fermion content changes. The values of the thresholds are contained in an array threshold(0:8). The threshold(1:7) entries are active thresholds, while threshold(0) is set to zero and threshold(8) to an arbitrary large number (currently $10^{200}$). For a given *Q*, the code identifies the index i such that threshold(i-1)$<Q<$ threshold(i). The flavour content at a given *Q* is then accessible through the integer array nflav(3,0:n_thresholds), where the nflav(1:3,i) entries indicate respectively the number of leptons, down-type, and up-type quarks at a given *Q*. The nflav(:,:) array is then used to compute the $\beta _{0,\mathrm QED}$ function b0_values(1:n_thresholds) at the seven threshold values and this is finally used to compute the value of the QED coupling alpha_values(1:n_thresholds) at the threshold values. The function Delete(qed_coupling) is also provided for consistency with general hoppet conventions, although in this case it does nothing. After this initialisation, the function Value(qed_coupling,mu) returns the QED coupling at scale $\mu$.

*QED splitting matrices*

The QED splitting matrices are stored in the object 
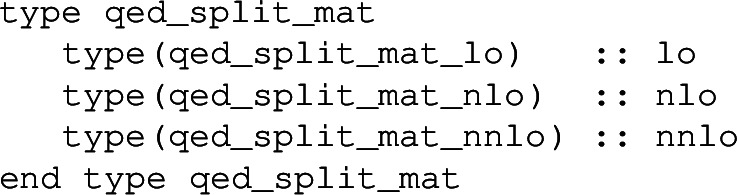


defined in qed_objects.f90. This contains the LO, NLO and NNLO splitting matrices 
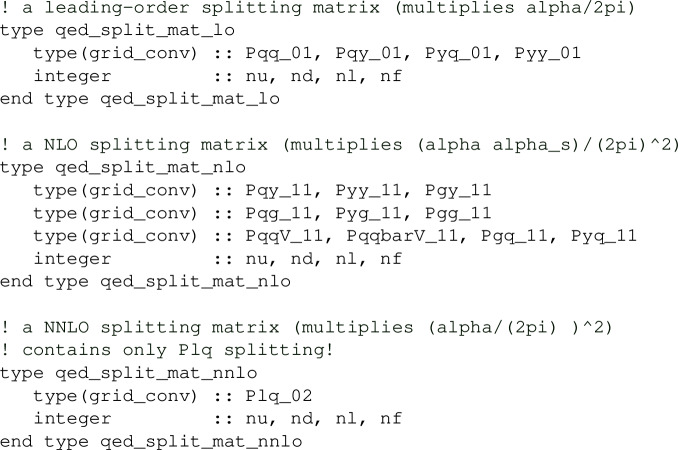


Above, y denotes a photon and the pairs of integers 01, 11 and 02 denote the orders in the QCD and QED couplings, respectively. Besides the number of quarks nf, these splitting matrices also need the number of up-type (nu) and down-type quarks (nd) separately, and the number of leptons (nl). Note that the splitting functions of order $\alpha$ (i.e. 01) for the leptons are simply obtained from the ones involving quarks by adjusting colour factors and couplings.

A call to the subroutine 
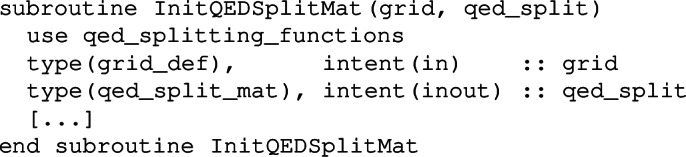


initializes the qed_split_mat object qed_split and sets all QED splitting functions on the given grid. The above QED objects can be used for any sensible value of the numbers of flavours, on the condition that one first registers the current number of flavours with a call to 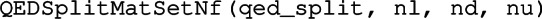


where nl, nd and nu are respectively the current numbers of light leptons, down-type and up-type quarks. In practice, this is always handled internally by the QED-QCD evolution routines, based on the thresholds encoded in the QED coupling.^13^ The one situation where a user would need to call this routine directly is if they wish to manually carry out convolutions of the QED splitting functions with a PDF.

Subroutines Copy and Delete are also provided for the qed_split_mat type. As in the pure QCD case, convolutions with QED splitting functions can be represented by the .conv. operator or using the product sign *.

*PDF arrays with photons and leptons*

A call to the subroutine 
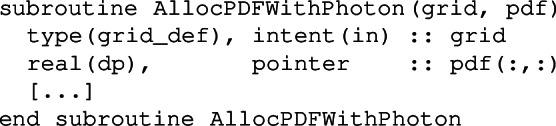


allocates PDFs (pdf) including photons, while a call to 
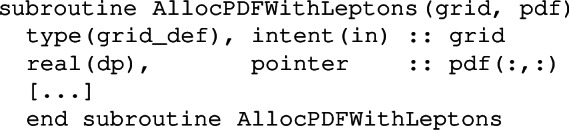


allocates PDFs including both photons and leptons. The two dimensions of the pdf refer respectively to the index of the *x* value in the grid, and to the flavour index. The flavour indices for photons and leptons are given by 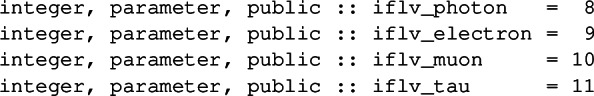


where each pdf(:,9:11) contains the sum of a lepton and anti-lepton flavour (which are identical). Note that if one were to extend the calculation of lepton PDFs to higher order in $\alpha$, then an asymmetry in the lepton and anti-lepton distribution would arise, due to the Plq_03 splitting function. In fact, at that order, there are also graphs with three electromagnetic vertices on the quark line and three on the lepton line, that change sign if the lepton line is charge-conjugated.^14^ In that case it would become useful to have separate indices for leptons and anti-leptons.

The subroutine AllocPDFWithPhotons allocates the pdf array with the flavour index from -6 to 8, while in the subroutine AllocPDFWithLeptons, the flavour index extends from -6 to 11.

*PDF tables with photons and leptons*

Next one needs to prepare a pdf_table object forming the interpolating grid for the evolved PDF’s. We recall that the pdf_table object contains an underlying array pdf_table%tab(:,:,:), where the first index loops over *x* values, the second loops over flavours and the last loops over $Q^2$ values.

This is initialized by a call to 
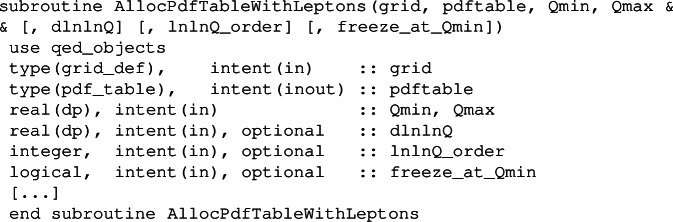


that is identical to the one without photon or leptons, the only difference is that the maximum pdf flavour index in pdf_table%tab now includes the photon and leptons. An analogous subroutine AllocPdfTableWithPhoton includes the photon but no leptons.

*Evolution with photons and leptons*

To fill a table via an evolution from an initial scale, one calls the subroutine 
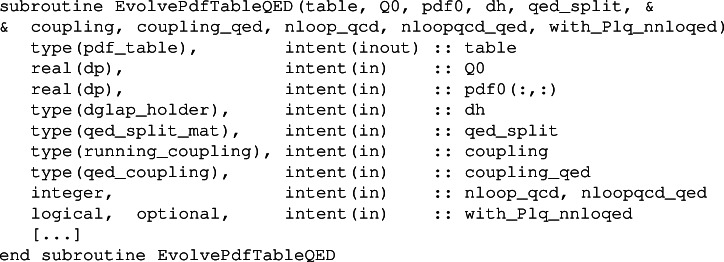


where table is the output, Q0 the initial scale and pdf0 is the PDF at the initial scale. We recall that the lower and upper limits on scales in the table are as set at initialisation time for the table. When nloopqcd_qed is set to 1 (0) mixed QCD-QED effects are (are not) included in the evolution. Setting the variable with_Plq_nnloqed=.true. includes also the NNLO $P_{lq}$ splittings in the evolution.

To perform the evolution EvolvePdfTableQED calls the routine 
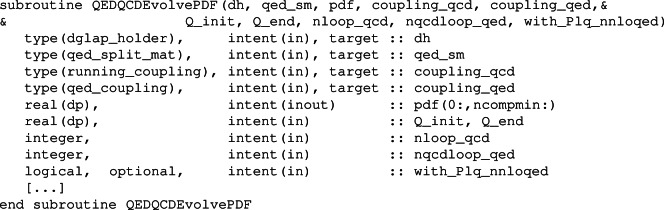


Given the pdf at an initial scale Q_init, it evolves it to scale Q_end, overwriting the pdf array. In order to get interpolated PDF values from the table we use the EvalPdfTable_* calls, described in Section 7.2 of Ref. [1].

However the pdf array that is passed as an argument and that is set by those subroutines should range not from (-6:6) but instead from (-6:8) if the PDF just has photons and (-6:11) if the PDF also includes leptons.^15^

Note that at the moment, when QED effects are included, cached evolution is not supported.

## Python interface

From version 2.0.0, hoppet also includes a Python interface to the most common evolution routines. It currently includes exactly the same functionality as the streamlined interface, and can be called in much the same way. The names of functions and routines are the same as in the streamlined interface, but stripped of the hoppet prefix. The interface can be obtained from PyPi by invoking 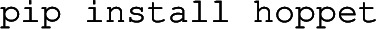


Alternatively the interface can be built by CMake with-DHOPPET_BUILD_PYINTERFACE=ON (cf. Sect. 8 for details on building with CMake). In both cases the interface can be imported into a Python instance through import hoppet. For a simple tabulation example, the user should take a look at examples/python/tabulation_example.py (and in the same directory for a number of other illustrative examples of how to use the interface, including in conjunction with LHAPDF [108]). The interface uses SWIG (Simplified Wrapper and Interface Generator) which therefore needs to be available on the system if building with CMake. It can be installed through most package managers (e.g. apt install swig, brew install swig) but can also be obtained from the SWIG GitHub https://github.com/swig.

One significant difference between the Python interface and the streamlined interface is that Python does not provide native support for pointers. A number of C**++**routines fill an array of PDF flavours that is passed as a pointer argument. Instead in Python, those routines return a Python list directly. For instance, where in C**++** one might have the call 
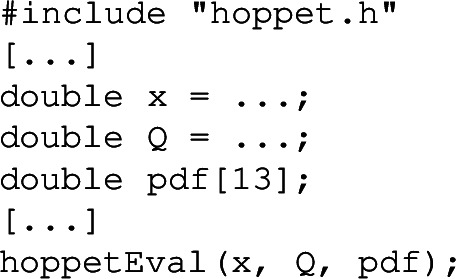


instead in Python one would have 
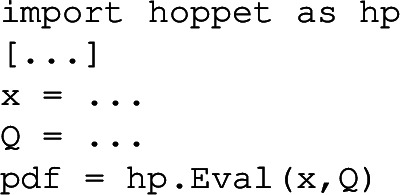


This is relevant not just for evaluation of PDFs, but also in setting initial conditions. For example the Assign, CachedEvolve, and Evolve routines should be passed a function of x and Q that returns an object that corresponds to array of flavours (it can be a numpy [115] array of a Python list; see the hera_lhc(x,Q) function in tabulation_example.py for an explicit example).

In addition to the examples provided in examples/python/ we have also developed a small tool that loads a grid from LHAPDF at an initial scale and evolves it with hoppet over a large range of *Q*. The resulting grids are then compared between hoppet and LHAPDF to determine the relative accuracy of the LHAPDF grids. The tool, along with some documentation, can be found at https://github.com/hoppet-code/hoppet-lhapdf-grid-checker.

## CMake build system

In v1.x, hoppet used a hand-crafted ./configure script followed by make [install]. As of v2 hoppet uses CMake.^16^

For a typical user it will be enough to invoke the following lines from the main directory 
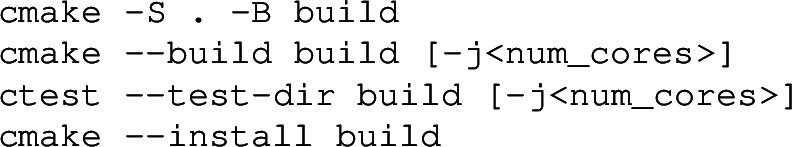


This will compile and install hoppet, along with the streamlined interface. Note that cmake –install build will typically install in a location that requires root privileges, unless a user has specified a custom prefix (through-DCMAKE_INSTALL_PREFIX=/install/path). A number of options can be passed to CMake. They are documented in CMakeLists.txt and can be printed on screen by a call to cmake -LH.. from the build directory.

Of particular note to most users are 
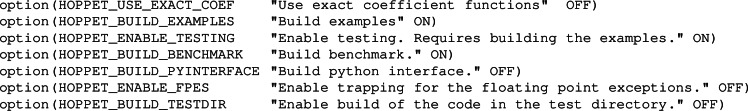


They can be set in the usual cmake way, e.g. 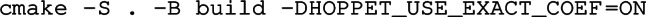


to compile hoppet with the exact coefficient functions. Note that the Python interface is not compiled by default, because we anticipate that users of Python will prefer to obtain hoppet through pip install hoppet.

Users should be aware that this release of hoppet makes use of features introduced in Fortran 2008, for example its abstract interface, and hence a Fortran 2008 compliant compiler is now needed. The code has been tested to compile and run with gfortran v10.5.0 and later, and the 2025 version of the Intel compiler ifx. If multiple Fortran compilers are available, a specific one can be chosen with the-DCMAKE_Fortran_COMPILER=... option.

## Saving LHAPDF grids

A minor new feature of this release is the possibility to save a hoppet table in the form of an LHAPDF6 [108] grid. The main routine has the following structure 
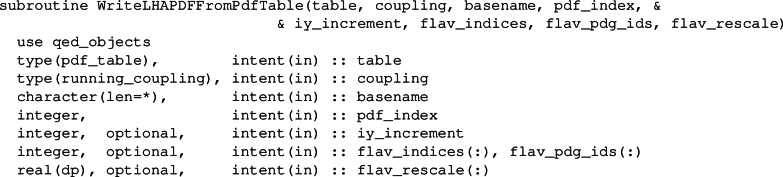


A user has to provide a table and associated coupling object along with a string basename and the pdf_index as needed by LHAPDF. If pdf_index is equal to 0 then the routine outputs the contents of the table in basename_0000.dat and writes a template basename.info again in LHAPDF format. hoppet fills most of the entries in the .info file, but a few need to be edited manually by the user. For any other value of pdf_index, only the corresponding .dat file gets written.

By default, the code uses the same grid spacing as in the internal hoppet table (iy_increment = 1) and prints all the possible flavours of the PDF, even if they are zero. The user can overwrite this default behaviour by providing an array of flavours and their pdg values. If iy_increment> 1 a coarser grid is provided by skipping over iy_increment - 1 points in the grid. The flav_rescale is currently needed for the lepton PDF which is provided as the sum over flavour and anti-flavour and therefore needs an extra factor half. The flav_rescale array only needs to be provided if the user is also providing the array of flavours and pdf values.

Finally the routine can also be accessed from the streamlined interface for C**++** or Python usage. In this case the routine is significantly simplified and only takes basename and the pdf_index arguments, for instance in C**++** like this 
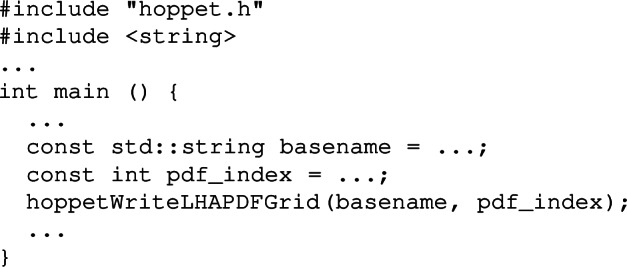


The routine writes the contents of the streamlined interface tables(0) and hence requires that this object has been filled either through a call to hoppetAssign or hoppetEvolve.

## Updated performance studies

In this section we present some updated performance studies relative to Section 9 of Ref. [1], mainly reflecting updated hardware and compilers of 2025, but also the standard nested grid choice that is obtained with the streamlined interface or, from the modern Fortran interface by calling InitGridDefDefault(grid, dy, ymax[, order]), with the default choice of interpolation order=-6.

The InitGridDefDefault(...) routine is new relative to v1 and sets up the following grid 
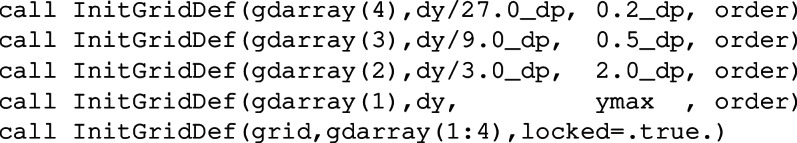


Users will usually only need a different choice if they plan studies at *x* very close to 1 or if they wish to explore fine optimisation of grid choices.

We split our study here into two parts: the accuracy of PDF evolution and tabulation (Sect. 10.1) and PDF evaluation (Sect. 10.2). The latter also outlines new functionality for choosing interpolation orders differently in the PDF evaluation versus the PDF evolution and it includes comparisons to LHAPDF.

### PDF evolution and tabulation

**Fig. 3:**
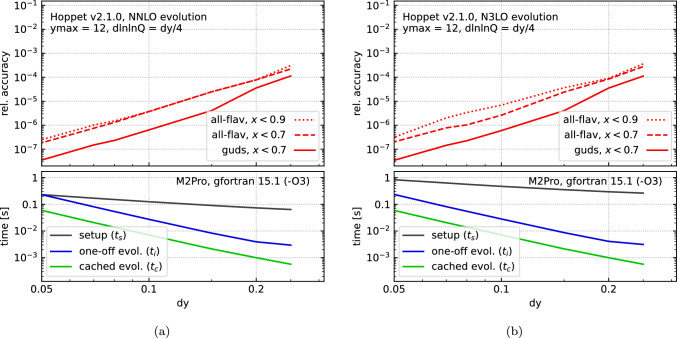
Accuracy (top) and timing (bottom) versus dy at (a) NNLO and (b) N$^3$LO in hoppet v2.1.0. The accuracy corresponds to the worst fractional accuracy for any flavour, at any *x* value below the corresponding limit, as described in the text. The timings were obtained on an M2Pro with gfortran v15.1 and-O3 optimisation. We have further used ymax = 12 and dlnlnQ = dy/4

The studies are performed using the initial condition of Ref. [8] as detailed in Section 9.1 of Ref. [1], evolved in the VFNS scheme. At NNLO we use the parametrised splitting functions and mass thresholds, as per hoppet defaults. To assess the accuracy we first create a reference run with a very high density grid. We then run hoppet with different values of grid spacing dy, keeping dlnlnQ = dy/4. The accuracy is then computed by looking at the largest relative deviation from the reference run across either all flavours (all-flav) or the light flavours (guds). The full tabulation covers the range $10^{-5}< x < 1$, $2< Q < 10^4\, \text {GeV}$.^17^ At *x* values close to 1, the numerical precision degrades because the parton distribution functions become a very steep function of $\ln x$. However, the parton distributions have small values there and so we carry out our precision study with two potential upper limits on the *x* value being probed, $x < 0.9$ and $x < 0.7$. We also exclude PDF flavours in the *x* and *Q* vicinity of any sign change, as per Ref. [1].

The results for the accuracy study can be seen in the top panels of Fig. 3a, b at NNLO and N$^3$LO respectively, as a function of dy. At NNLO, the accuracy comes out similar to previous versions of hoppet. With dy = 0.2 one obtains a relative accuracy of $10^{-4}$ across all flavours in the range $x < 0.9$. At the finest grid spacing dy = 0.05, a relative accuracy of few times $10^{-7}$ can be achieved, good enough for precise benchmark comparisons as were for instance carried out in Refs. [8, 9]. For comparison, the recent benchmark of aN$^3$LO codes in Ref. [97] reaches a relative precision of a few times $10^{-4}$ at best (cf. the gluon PDF at $x = 10^{-2}$ in Table 5 and Table 1 of Ref. [97] which differ by $\sim 8\cdot 10^{-4}$.)

The scaling of the precision is roughly consistent with a power law in dy. In particular the Runge–Kutta algorithm for the $Q^2$ evolution is expected to yield an error proportional to $\texttt {dlnlnQ}^4$, which, given our choice of $\texttt {dlnlnQ} = \texttt {dy}/4$ translates into a behaviour $\sim \texttt {dy}^4$ in Fig. 3. The observed scaling is, if anything, slightly better than this, given the factor of 1000 improvement in accuracy when reducing dy by a factor of 5 from 0.2 to 0.04. The precise scaling depends on whether it is Runge–Kutta or the splitting function grid representation that dominates the error. At N$^3$LO we observe a similar level of accuracy as at NNLO, except for a slight worsening associated with the heavy-flavour components.

For the timing studies we again run hoppet for different values of dy, on an M2Pro (MacOS 15.6.1) with gfortran v15.1 and-O3 optimisation. As discussed in [1], the time spent in hoppet for a given analysis can be expressed as follows, depending on whether or not one carries out cached evolution (pre-evolution):

11a $$\begin{aligned} t_\text {no pre-ev}&= t_s + n_\alpha t_\alpha + n_i (t_i + n_{xQ}\, t_{xQ})\,, \end{aligned}$$

11b $$\begin{aligned} t_\text {with pre-ev}&= t_s + n_\alpha (t_\alpha + t_p) + n_i (t_c + n_{xQ}\, t_{xQ})\,, \end{aligned}$$

where $t_s$ is the time for setting up the splitting functions and mass threshold functions, $n_\alpha$ is the number of different running couplings that one has, $t_\alpha$ is the time for initialising the coupling, $n_i$ is the number of PDF initial conditions that one wishes to consider, $t_i$ is the time to carry out the tabulation and evolution for a single initial condition, $n_{xQ}$ is the number of points in *x*, *Q* at which one evaluates the full set of flavours once per PDF initial condition; in the case with cached evolution, $t_p$ is the time for preparing a cached evolution and $t_c$ is the time for performing the cached evolution. Finally, $t_{xQ}$ is the time it takes to evaluate the PDFs at a given value of (*x*, *Q*) once the tabulation has been performed.

Here we focus on $t_s$, $t_i$, and $t_c$. The results can be seen in the bottom panels of Fig. 3a, b at NNLO and N$^3$LO respectively. The expected scaling is $t_i, t_c \sim ({\texttt {dy}}^2 {\texttt {dlnlnQ}})^{-1}$, which for our choice of $\texttt {dlnlnQ} \propto \texttt {dy}$ reduces to $1/{\texttt {dy}}^3$. That is consistent with what is seen in the plot. Turning to the setup time, at NNLO $t_s \sim 60-300\,\textrm{ms}$ dominates over the evolution time across almost all dy values that we study. It scales slightly more slowly than $t_s \sim 1/\texttt {dy}$.

We note that when using cached evolution, the evolution time $t_c$ reaches as little as $1\,\textrm{ms}$ for dy = 0.2. Comparing these numbers to those of Table 2 of Ref. [1], which were obtained with 2008 hardware and compilers, we see a speed-up of roughly a factor 10, which we attribute mainly to improvements in the hardware.

At N$^3$LO evolution times ($t_i$ and $t_c$) are essentially identical to the NNLO case: for $t_i$ the only extra operation that is needed is the addition of the N$^3$LO splitting function to the lower-order ones and at each *Q* value, this involves ${{\mathcal {O}}}\left( N\right)$ operations for a *y*-grid of size *N*, while the convolution itself involves ${{\mathcal {O}}}\left( N^2\right)$ operations. For the cached evolution, there is no additional penalty, because the N$^3$LO contributions are already included in the cached evolution operators. The initialisation times are somewhat larger, in a range from 250 ms to 1 s. The longer time is associated both with the approximate N$^3$LO splitting functions and the mass threshold functions of Ref. [85], using the n3lo_nfthreshold_libOME option. In practice the initialisation time remains adequate for most interactive work. In any long-running application where hoppet either has to evolve or access the evolved tables many times, the initialisation time is insignificant.

### Fast PDF access

Here we detail updates for faster PDF access within the modern Fortran and streamlined interfaces (including the Python interface). In earlier versions of hoppet, the interpolation was carried out by a single routine that could flexibly handle any choice of *y* and *Q* interpolation orders up to some hard-coded maximum. As of v2.0.0, a number of interpolation-order choices now have dedicated code, which makes it easier for the compiler to optimise the underlying assembly, e.g. with loop unrolling, giving speed gains of almost a factor of three. Additionally new functionality allows the user to modify the interpolation order of the hoppet grids, trading accuracy versus speed.

Specificaly, the user can now globally override any table-specific interpolation order settings by calling one of 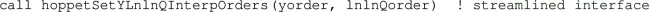


or 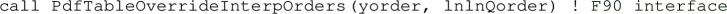


A value of $\texttt {order}=2$ corresponds to quadratic interpolation, 3 to cubic interpolation, etc.^18^ The default interpolation order is quartic in the lnlnQ direction, and |grid%order|-1 in the y direction (bounded to be between 3 and 9 if outside that range). These rather high interpolation orders help ensure good accuracy in a normal hoppet run even with $\texttt {dy=0.2}$, but come with a speed penalty because of the larger number of operations. However, if PDF evaluation represents a significant fraction of the time for a user’s code, the user can choose to lower the interpolation order and still retain good accuracy by reducing dy and dlnlnQ.

**Fig. 4:**
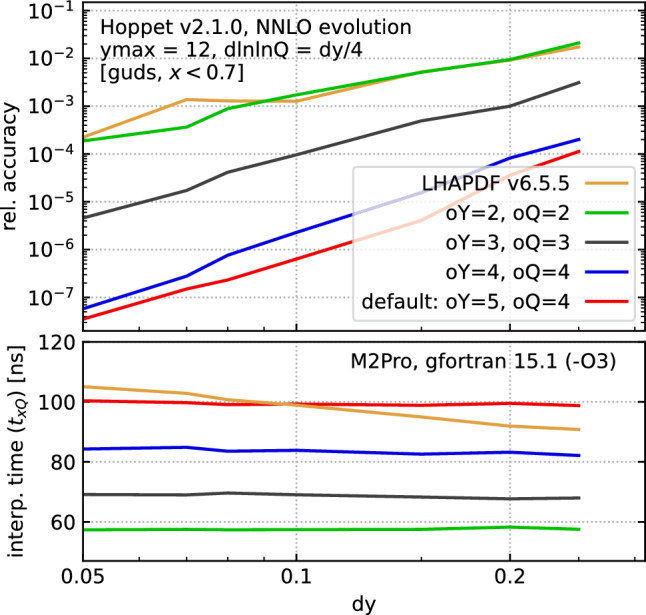
Study of the impact of the choice of the *y* and *Q* interpolation orders (oY, oQ) on accuracy (upper panel) and speed (lower panel) for evaluating the PDF table at a given *y* and *Q* point. Note that the interpolation order that enters the splitting functions remains 6 as in Fig. 3. The timings correspond to the EvalPdfTable_yQ(table,y,Q,vals) modern Fortran call. The timings have been obtained on an M2Pro with gfortran v15.1 and-O3 optimisation. Calling EvalPdfTable_xQ(table,x,Q,vals), or the corresponding functions in the streamlined interfaces, adds about $5\;\textrm{ns}$. We have further used ymax = 12 and dlnlnQ = dy/4. Note that our accuracy definition is to show the *worst* accuracy across any [*guds*] flavour and *x* value in range. In practice, most points have a significantly higher accuracy. We also show results obtained by interpolating our grids with LHAPDF 6.5.5, calling it from its native C**++**interface to maximise its speed

**Table 6:** Time for a call to hoppetEval, which evaluates all flavours at a given *x*, *Q* point. The rows show the timings with different interpolation orders. The results have been obtained with the PDF4LHC21_40 set, on an M2Pro with gfortran−15.1, Apple clang version 17.0.0, O3 optimisation and Python 3.12.7. The hoppet grid spacings are $\texttt {dy}=0.05$ and $\texttt {dlnlnQ}=\texttt {dy}/4$, with $\texttt {ymax} = 14$. The table also shows the timings for the equivalent calls in LHAPDF 6.5.5. Version 6.5.5 of LHAPDF, in its Fortran (EvolvePDF(...), lhapdf_xfxq(...)) and Python (pdf.xfxQ(...)) interfaces, loops over an underlying call to single-flavour evaluation (double PDF::xfxQ(int id, double x, double q))), which is why it is significantly slower than the C**++**interface, which evaluates all flavours in one go. A proposed patch for the Fortran and Python interfaces has been submitted to LHAPDF with corresponding timings also indicated

yorder	lnlnQorder	Time per hoppetEval or LHAPDF xfxQ/EvolvePDF call (ns)		
		Fortran	C**++**	Python
5	4	108	108	295
4	4	92	93	278
3	3	76	77	258
2	2	63	63	244
HOPPET 1.2.0		313	313	–
LHAPDF 6.5.5		520	87	1645
LHAPDF 6.5.5 + patch		114	87	1028

Figure 4 shows the accuracy and timing as a function of dy for different interpolation order choices. It compares evolution as in Fig. 3, followed by interpolation with a given order. It illustrates the significant loss in accuracy when decreasing the interpolation order below 4. For orders of 4 or higher, the limitation on accuracy is, however, no longer just the interpolation order during PDF evaluation, but also the orders that appear in the grid representation of the splitting functions and of the *Q* evolution when producing the original table.^19^ The time that is shown in the lower panel corresponds to the evaluation of all flavours in one go, via the EvalPdfTable_yQ(...) routine. It ranges from $60\;\textrm{ns}$ to $100\;\textrm{ns}$ in going from the (2, 2) order combination to (5, 4). It is largely independent of dy. Calls to evaluate a single flavour are only $2.5{-}3$ times faster, because of overheads associated with identifying the grid location and calculation interpolation coefficients, which are independent of the number of flavours one evaluates.

Figure 4 also shows results with LHAPDF, calling it from its native C**++**to maximise its speed. We generate an LHAPDF grid for our standard benchmark initial conditions, with a given dy spacing, cf. Sect. 9, and then examine the difference between the LHAPDF evaluation and our high-accuracy reference. The upper panel of Fig. 4 shows the accuracy with the same definition as used in our other performance results, i.e. the worst relative accuracy observed anywhere for $x < 0.7$, across any of the g, u, d, s flavours, as in the solid lines of Fig. 3. For most flavours and most of the *x* region, the accuracy is better than shown. Interestingly, the LHAPDF accuracy is comparable to hoppet ’s quadratic interpolation. At first sight this might be surprising given that LHAPDF uses cubic interpolation: however it is our understanding that in the *x* direction it uses a cubic *spline*. The spline effectively sacrifices one of the orders of accuracy in function evaluation and instead uses it to ensure exact continuity of the first derivative. In contrast, hoppet does not enforce continuity of the derivatives but relies on the fact that for high interpolation order any discontinuity of the derivatives will scale as a high power of the grid spacing. Which choice is better may depend on the application. Concerning speed, we find that LHAPDF is somewhere in between our (4, 4) and (5, 4) choices.

For use-cases where PDF evaluation speed is critical, one option is to read in a PDF grid with LHAPDF, evaluate it at all hoppet grid points and then use hoppet for the interpolation, using a fine grid spacing and a (2, 2) interpolation choice. We provide examples for how to do this in Fortran, C**++**, and Python:

- https://github.com/hoppet-code/hoppet/blob/hoppet-2.1.1/examples/f90/with-lhapdf/: it uses the module hoppet_lhapdf and the associated subroutine LoadLHAPDF(name, imem). The module is in the same directory and can be copied over to a user’s application;
- https://github.com/hoppet-code/hoppet/blob/hoppet-2.1.1/examples/f90/withlhapdf/lhapdf_to_hoppet_allmembers.f90, same as above but the example shows how to read in and efficiently evaluate all LHAPDF members, with an illustration for computing the PDF uncertainty;
- https://github.com/hoppet-code/hoppet/blob/hoppet-2.1.1/examples/cpp/with-lhapdf/lhapdf_to_hoppet.cc, which includes a routine called void load_lhapdf_assign_hop pet(const string & pdfname, int imem=0) which can be included directly in a user’s application;
- https://github.com/hoppet-code/hoppet/blob/hoppet-2.1.1/examples/python/lhapdf_to_hoppet.py, makes use of hoppet.lhapdf.load(), accessible using “from hoppet import lhapdf”.

The PDF can then be evaluated in the usual way through a call to hoppetEval and likewise for the coupling through hoppetAlphaS.^20^ All three examples also print the timings to the screen so that users can check speed on their hardware. Note that these examples are not fully general, and caution should be exercised when using them. Users with more advanced requirements are invited to contact the hoppet authors for assistance.

Table 6 illustrates those timings for a range of interpolation orders and across interfaces in different languages. These tests have been carried out with the PDF4LHC21_40 set. Again, they confirm that hoppet with lower interpolation orders can offer a speed gain relative to LHAPDF. That speed gain is moderate in C**++**, more significant in Fortran and Python. As concerns Fortran, there are straightforward modifications to LHAPDF that would improve its speed and these have been proposed to the LHAPDF authors.

The PDF and $\alpha _s$ evaluation routines in hoppet are thread safe. Other parts of the code, notably initialisation and evolution, are not.

## Conclusion

Version 2 of hoppet brings major additions to its functionality. These include evolution up to N$^3$LO, massless structure function evaluation, QED evolution, a Python interface, a modern build system, functionality for writing LHAPDF grids and significant speed improvements in the interpolation of its internal PDF tables.

Overall hoppet remains highly competitive in terms of speed and accuracy. For example, repeated filling of a full PDF tabulation takes about a millisecond per initial condition, with a relative accuracy of $10^{-4}$ or better for $10^{-5}< x<0.9$. It offers explicit handles to control the accuracy, allowing users to verify the precision of their results and choose the optimal trade-off between speed and precision. Its modern Fortran interface also offers powerful and flexible access to a range of common PDF manipulations such as convolutions with arbitrary splitting and coefficient functions, features that are useful in a variety of contexts.

We hope that this release of hoppet can help provide solid foundations for a range of groups to contribute to the ongoing discussions [49, 50, 91, 97, 116] in the field concerning the impact of N$^3$LO and QED effects in PDF fits. We also hope that the interfaces across computing languages will facilitate the practical aspects of integration with a range of other tools.

## Data Availability

My manuscript has associated code/software in a data repository. [Author’s comment: No data was generated as this is a pure code/theory study.]

## Footnotes

1 Other codes such as ChiliPDF [7] appear to not yet be public.

2 The general structure of the relation between space-like and time-like evolution and splitting functions has been investigated in [39, 40, 63–68]. See references to those articles for more recent updates.

3 In a general scheme they would start at NLO.

4 Note that the literature focuses on the $q_{l,i}+q_{l,-i}$ combination. We thank Johannes Blümlein for having provided the $A^{\mathrm{NS,-}}_{qq,h}(x)$ code needed for the $q_{l,i}-q_{l,-i}$ combination.

5 We thank the authors for the code corresponding to the calculation.

6 We thank Johannes Blümlein for sharing a pre-release version the code from Ref. [85] with us, which also contains code associated with Refs. [82, 84].

7 The implementation does not allow for non-standard values of the QCD Casimir invariants beyond three loops.

8 Note that the piece presented in Ref. [106] has not been given in exact form. Only a parametrised version is available and is what is being used in hoppet.

9 Users needing a lower level interface should inspect the code in https://github.com/hoppet-code/hoppet/blob/hoppet-2.1.1/src/structure_functions.f90.

10 The LO and NLO coefficient functions are always exact as their expressions are very compact. Note that for very large values of *x* we switch to a large-*x* expansion for the regular part of non-singlet coefficient functions at N$^3$LO, to avoid numerical instabilities in the exact expressions as discussed in Appendix A of Ref. [9].

11 The above counting is to be contrasted with a “democratic” scheme, in which one considers $\alpha \sim \alpha _s\sim 1/L$ and where the aim would be to maintain the same loop order across all couplings and splitting functions, regardless of the relative numbers of QCD and QED couplings that they involved.

12 The implementation of the 2-loop $P_{\ell q}$ splitting function in the hoppet code (Plq_02 in the code) was carried out by Luca Buonocore.

13 While the QCD splitting functions are initialised and stored separately for each relevant value of $n_f$, in the QED case the parts that depend on the numbers of flavours are separated out. Only when the convolutions with PDFs are performed are the relevant $n_f$ and electric charge factors included.

14 Analogous terms appear in the non-singlet three-loop splitting functions [41].

15 Index 7 is reserved in hoppet to hold information about the flavour representation of PDFs. Ideally PDFs with the full set of flavours would be represented by an underlying type with a 2D array for storing the *x*-dependence of the different PDFs, supplemented with an index for the representation. However when we explored this option during v1 development we found that this compromised our ability to maintain speed while writing operations of PDFs such as a=b+d*c We thus opted to work with plain 2D arrays, encoding the flavour structure with a specific signature for the *x*-dependence of index 7. This is not ideal, however changing it would come at the price of breaking backwards compatibility, and one would still need to explore whether, with today’s Fortran features, one could find a good solution without a speed impact.

16 We thank Andrii Verbitskyi for providing much of this system. Support for the old build system was retained in the briefly lived 2.0.x series, but was retired in version 2.1.0 owing to the need to support compilation with both Fortran and C**++**, the latter for the libome library.

17 For tabulations extending to significantly smaller *x* values, it can be advantageous to take a smaller dlnlnQ choice, e.g. dlnlnQ = dy/8, because of the steep $\ln Q$ derivative of the parton distributions at the smallest *x* values.

18 The (yorder, lnlnQorder) choices with dedicated code are (2, 2), (3, 3), (4, 4), (5, 4) and (6, 4).

19 Recall that the former can be controlled with the order argument when setting up the grid. Currently, the latter is hard-coded as part of the Runge–Kutta evolution routines (cf. above).

20 For the PDF, hoppet just interpolates its table, which is filled directly from LHAPDF. For $\alpha _s$, hoppet carries out its own evolution, which may have small differences from the LHAPDF $\alpha _s$, notably if the LHAPDF grid provides a coupling that is not an exact solution of the renormalisation group equation. Another subtlety concerns the top threshold. The LHAPDF grid files usually quote a physical top mass, and in the examples above, hoppet reads the top mass and provides $\alpha _s$ evolution at higher scales with 6 light flavours. However in some LHAPDF sets, the top-mass is only intended to indicate the value of the top mass used in the PDF fit, not an $n_f = 5 \rightarrow 6$ threshold in the evolution. The examples above all use a call similar to if(!pdf->hasFlavor(6)) mt = 2.0*Qmax; which manually overrides the top mass to a value outside the grid ranges if there is no top quark present in the set.

## Code Availability

This manuscript has no associated data. [Author’s comment: The HOPPET code is available from the following git repository: https://github.com/hoppet-code/hoppet.]
